# Ethacrynic Acid Exhibits Selective Toxicity to Chronic Lymphocytic Leukemia Cells by Inhibition of the Wnt/β-Catenin Pathway

**DOI:** 10.1371/journal.pone.0008294

**Published:** 2009-12-14

**Authors:** Desheng Lu, Jerry X. Liu, Tomoyuki Endo, Haowen Zhou, Shiyin Yao, Karl Willert, Ingo G. H. Schmidt-Wolf, Thomas J. Kipps, Dennis A. Carson

**Affiliations:** 1 Moores Cancer Center, University of California San Diego, La Jolla, California, United States of America; 2 Department of Cellular and Molecular Medicine, University of California San Diego, La Jolla, California, United States of America; 3 Center for Integrated Oncology, University of Bonn, Bonn, Germany; Texas Tech University Health Sciences Center, United States of America

## Abstract

**Background:**

Aberrant activation of Wnt/β-catenin signaling promotes the development of several cancers. It has been demonstrated that the Wnt signaling pathway is activated in chronic lymphocytic leukemia (CLL) cells, and that uncontrolled Wnt/β-catenin signaling may contribute to the defect in apoptosis that characterizes this malignancy. Thus, the Wnt signaling pathway is an attractive candidate for developing targeted therapies for CLL.

**Methodology/Principal Findings:**

The diuretic agent ethacrynic acid (EA) was identified as a Wnt inhibitor using a cell-based Wnt reporter assay. *In vitro* assays further confirmed the inhibitory effect of EA on Wnt/β-catenin signaling. Cell viability assays showed that EA selectively induced cell death in primary CLL cells. Exposure of CLL cells to EA decreased the expression of Wnt/β-catenin target genes, including LEF-1, cyclin D1 and fibronectin. Immune co-precipitation experiments demonstrated that EA could directly bind to LEF-1 protein and destabilize the LEF-1/β-catenin complex. N-acetyl-L-cysteine (NAC), which can react with the α, β-unsaturated ketone in EA, but not other anti-oxidants, prevented the drug's inhibition of Wnt/β-catenin activation and its ability to induce apoptosis in CLL cells.

**Conclusions/Significance:**

Our studies indicate that EA selectively suppresses CLL survival due to inhibition of Wnt/β-catenin signaling. Antagonizing Wnt signaling in CLL with EA or related drugs may represent an effective treatment of this disease.

## Introduction

Chronic lymphocytic leukemia (CLL) is one of the most common hematological malignancies in the United State. Despite significant advances in the treatment of CLL and its complications, there is no cure for this disease. CLL is characterized by a progressive accumulation of morphologically mature but functionally incompetent lymphocytes in peripheral blood, secondary lymphoid tissue, and bone marrow [1]. However, it remains unclear how the clonal expansion of B-lymphocytes in CLL is caused by an imbalance between signals that promote cell survival and apoptosis [2], [3], [4]. The identification of molecular pathways that the malignant cells use for survival in CLL may thus provide novel potential targets for therapy.

Wnt signaling affects fundamental development pathways by regulating cell proliferation and differentiation. Aberrant activation of the Wnt signaling pathway has major oncogenic effects [5], [6], [7], [8], [9]. In the canonical Wnt pathway, the secreted Wnt proteins bind to a receptor complex, consisting of a member of the Frizzled (Fzd) family, and the low-density lipoprotein-receptor-related proteins (LRP) 5 or LRP6. Subsequently the cytoplasmic adaptor protein disheveled (Dvl) is phosphorylated and inhibits glycogen synthase kinase (GSK)-3β activity through its association with axin. Unphosphorylated β-catenin accumulates in the cytoplasm and translocates into the nucleus, where it interacts with T cell (TCF) and lymphoid-enhancing (LEF) factors to activate transcription of Wnt target genes [5], [6], [8]. Recently, it has been demonstrated that the Wnt signaling pathway is activated in CLL cells, and that uncontrolled Wnt/β-catenin signaling may contribute to the defect in apoptosis that characterizes this malignancy [10], [11]. In comparison to normal blood B cells, LEF-1 is the most highly upregulated mRNA in CLL cells [12]. The orphan Wnt receptor ROR1, whose promoter contains multiple LEF-1 regulatory motifs, is also highly expressed in CLL. Thus, the Wnt signaling pathway, and especially LEF-1, are attractive candidates for developing targeted therapies for CLL.

Ethacrynic acid (EA), a once commonly used loop diuretic drug, was previously shown to be cytotoxic toward primary CLL cells [13], [14] and other tumor cells [15], [16]. The mechanism of EA cytotoxicity was attributed to the drug's known capacity to inhibit glutathione S-transferase (GST), causing increased cellular oxidative stress. However, a recent study [17]showed that the antioxidant N-acetyl-L-cysteine (NAC) protected cells from EA-induced apoptosis with no effect on cellular glutathione (GSH) levels, whereas the free radical scavenger 3-*t*-butyl-4-hydroxyanisole (BHA) did not, suggesting the existence of additional or alternative pathways that are altered by the drug. In a previous study, we prepared a series of EA analogs, and showed that their cytotoxicity to CLL cells depended upon the α, β-unsaturated ketone in the molecules, that can react with cellular thiols by Michael addition [18].

Here we demonstrate that EA can directly interact with LEF-1 protein in CLL cells, and destabilize the LEF-1/β-catenin complex. Importantly, EA exhibited selective cytotoxicity towards primary CLL cells, possibly due to the dependence of the malignant lymphocytes on proteins regulated by LEF-1 transcription.

## Materials and Methods

### Human Samples

Blood samples were collected by the Chronic Lymphocytic Leukemia Research Consortium, after obtaining informed consent from patients fulfilling diagnostic criteria for CLL, at all disease stages. Institutional review board approval was obtained from University of California San Diego (Approval#080918) for the procurement of patient samples in this study, in accordance with the Declaration of Helsinki. The patients in this study have given written informed consent to publication of their case details.

### Chemical Reagents

Ethacrynic acid (EA), *N*-acetyl-L-cysteine (NAC), pyrrolidinedithiocarbamate ammonium salt (PDTC), and 3-*t*-butyl-4-hydroxyanisole (BHA) were from Sigma-Aldrich (St. Louis, MO). A Gen-plus collection of 960 known drugs was obtained from Microsource (Gaylordsville, CT).

### Transfection and Screening of Drug Library

The human embryonic kidney cell line HEK293 (American Type Culture Collection, Rockville, MD) was transfected using the FuGene transfection reagent (Roche Diagnostics GmbH, Mannheim, Germany) according to the manufacturer's instruction.

The reporter plasmids TOPflash and FOPflash were gifts from H. Clevers (University of Utrecht, Utrecht, The Netherlands). The pNFAT-Luc reporter was purchased from BD Biosciences. The expression plasmids encoding Wnt1, Wnt3, LRP6, Dvl, β-catenin and NFATc have been described previously [10], [19].

For screening of the drug library, HEK293 cells were grown for at least 24 h in 10 cm plates prior to transfection. At ∼50% confluence, cells were transfected with 5 µg of TOPflash reporter, 1 µg expression vector for Dvl, 1 µg of control plasmid pCMXβgal and carrier DNA pcDNA3 plasmid for a total of10 µg/plate. After transfection for 24 h, cells were harvested and dispersed in 96-well microtiter plates. Then the cells were treated with the different agents, generally at 10 µM and 50 µM for the initial screen. After overnight incubation, the cells were lysed in 1x potassium phosphate buffer, pH 7.8, containing 1% Triton X-100, and luciferase activities were assayed in the presence of substrate using a microtiter plate luminometer (MicroBeta TriLux, Gaithersburg, MD). The luciferase values were normalized for variations in transfection efficiency using the β-galactosidase internal control. EA, and other compounds that were scored positive, had ≥30% inhibition of TOPflash activity when compared to the designated control cultures. In other experiments, transient transfections were performed in 12-well plates. HEK293 or SW480 cells were transfected with 0.5 µg of reporter plasmid, 0.1 µg of control plasmid pCMXβgal, 0.1–0.2 µg expression plasmids, and carrier DNA pcDNA3 plasmid for a total of 1 µg/well. After 16 h, the cells were washed and treated with 50 µM EA or solvent (DMSO) for 24 h. Then luciferase values were determined. In the Results section, data are expressed as fold stimulation of luciferase activity compared to the basal level. All the transfection results represent means of a minimum of three independent transfections assayed in duplicate, ±the standard error of the mean (SEM).

### Activation of TOPflash Reporter Using Wnt3a

The Super8XTOPflash construct (kindly provided by Dr. R. Moon) was stably transfected into HEK293 cells, and single cell clones were isolated. The stable Super8XTOPflash reporter cell line displays low basal luciferase activity and strong luciferase induction in response to Wnt3a stimulation. Preparation of Wnt3a and Wnt3a stimulations were performed as described [20], [21].

### Cell Viability Assay with 3-[4,5-Dimethylthiazol-2-yl]-2,5-diphenyl Tetrazolium Bromide (MTT)

Primary CLL cells were collected from patients' peripheral bloods after informed consent, and isolated by Ficoll/Hypaque density-gradient centrifugation as previously described [10]. Normal peripheral blood mononuclear cells were also purified as described [10]. The cells were resuspended in RPMI 1640 medium with 10% fetal bovine serum (Gemini Bio-Products, West Sacramento, CA), and antibiotics at 37°C, 5% CO_2_. Cell viability after drug exposure was assessed by MTT assay. Fresh CLL or peripheral blood mononuclear cells were plated at 2.5×10^5^ per well in 96-well plates. After 48 h, 1/10 volume of 5 mg/ml MTT was added, and cells were incubated at 37°C overnight. Finally, ½ volume of Lysis buffer was added to the cultures, and ODs at 570 nm were read and recorded.

### Cell Apoptosis Assays

The apoptosis of the CLL cells was determined by the analysis of mitochondrial transmembrane potential (ΔΨ_m_) using 3, 3′-dihexyloxacarbocyanine iodine (DiOC_6_) and by cell membrane permeability to propidium iodide (PI). Primary CLL cells were treated with 3 µM EA, 1 mM NAC, 100 µM BHA or combined treatment as indicated. After treatment for 48 h, the cells were stained with DiOC_6_ and PI and analyzed by flow cytometry. For each assay, 100 µl of the cell culture at a density of 10^6^ cells/ml was collected at the indicated time points and transferred to polypropylene tubes containing 100 µl of 60 nM DiOC_6_ and 10 µg/ml PI in FACS buffer containing serum deficient RPMI medium with 0.5% bovine serum albumin (BSA). The cells were then incubated at 37°C for 15 minutes and analyzed within 30 minutes by flow cytometry using a FACSCalibur (Becton Dickinson). Fluorescence was recorded at 525 nm (FL-1) for DiOC_6_ and at 600 nm (FL-3) for PI. The apoptotic cells were determined by calculating the percentages of the DiOC_6_^+^/PI^−^ CLL populations.

### RNA Isolation and Real-Time PCR

Primary CLL cells from three patients were treated with increasing amounts of EA for 16 h. Total RNA was isolated from 1×10^6^ CLL cells by Trizol reagent (Invitrogen, Carlsbad, CA). The RNA samples were further purified using a Qiagen RNeasy Protect kit (Qiagen, Valencia, CA). The mRNA levels were quantified in duplicate by real time PCR on the iCycler iQ detection system for TaqMan assay (Bio-Rad Laboratories, Hercules, CA) using the following primer sets: cyclin D1 forward 5′GGCGGAGGAGAACAAACAGA3′, reverse 5′ TGGCACAAGAGGCAACGA 3′ and probe 5′TCCGCAAACACGCGCAGACC 3′, Fibronectin forward 5′ACCTACGGATGACTCGTGCTTT3′, reverse 5′TTCAGACATTCGTTCCCACTCA3′ and probe 5′CCTACACAGTTTCCCATTATGCCGTTGGA 3′, Fzd5 forward 5′ CGCGAGCACAACCACATC3′, reverse 5′ AGAAGTAGACCAGGAGGAAGACGAT3′ and probe 5′ TACGAGACCACGGGCCCTGCAC3′. LEF-1 mRNA level was detected using TaqMan Gene Expression assay Hs00212390_m1 (LEF-1) (Applied Biosystems). PCR was performed using Taqman PCR Core Reagents (Applied Biosystems, Foster City, CA, USA) according to the manufacturer's instructions. PCR cycles consisted of an initial denaturization step at 95°C for 15 s and at 60°C for 60 s. PCR amplification of 18S RNA was done for each sample as a control for sample loading and to allow for normalization between samples. The data were analyzed using the comparative Ct method, where Ct is the cycle number at which fluorescence first exceeds the threshold. The ΔCt values from each cell line were obtained by subtracting the values for 18S Ct from the sample Ct. One difference of Ct value represents a 2-fold difference in the level of mRNA. The mRNA level was expressed as percentage with respect to control (100%).

### Preparation of Ethacrynic Acid Antiserum

A conjugate of EA with Keyhole Limpet Hemocyanin (KLH, Sigma) was prepared by thiolation of KLH with N-succinimidyl S-acetylthioacetate (SATA), followed by allowing the SATA-KLH conjugate to form a Michael adduct with EA, as described [22]. Immunization of rabbits was performed by three 1 ml subcutaneous injections of approximately 0.4 mg EA-KLH conjugates. Complete Freund's adjuvant was used for the first injection. The second and third injections were performed 3 and 6 weeks after the first, using incomplete adjuvant. The rabbits were bled six weeks after the third injection for preparation of antiserum. The specificity of the antibody was confirmed by both ELISA and immunoblotting using EA conjugated to a different antigen (ovalbumin).

### Co-Immunoprecipitation and Immunoblotting

Primary CLL cells and SW480 cells were treated with the indicated amounts of EA as described in the Figure legend. Cells were washed twice with PBS and resuspended in 0.5 ml lysis buffer (20 mM Tris-HCl, pH 8.0/10% glycerol/5 mM MgCl_2_/0.15 M KCl/0.1% Nonidet P-40 with protease inhibitors). For CLL cells, lysates of 1 to 2×10^7^ cells were incubated with anti-LEF-1 antibody at a 1∶1000 dilution overnight at 4°C, and then with saturating amounts of protein G plus/protein A agarose beads (Calbiochem) at 4°C for 2 h before centrifugation at 15,000 g for 5 min. For SW480 cells, lysates of 0.5 to 1×10^7^ cells were incubated overnight at 4°C with saturating amounts of agarose beads linked to monoclonal antibodies specific for β-catenin (Santa Cruz Biotechnology). The beads were washed twice with lysis buffer and once with PBS. Bound proteins were eluted by boiling the samples in SDS sample buffer and resolved by SDS/PAGE followed by immunoblotting with anti-EA antibody (1∶1000), anti-LEF-1 antibody (1∶1000) (BD Biosciences), anti-β-catenin antibody (1∶2000) (Santa Cruz Biotechnology), anti-α-catenin antibody (1∶2000) (GenWay). Horseradish peroxidase-conjugated anti-IgG was used as the secondary antibody. The membranes were developed using a chemiluminescence system (ECL detection reagent, Amersham Pharmacia Life Science). For some experiments, the immunoblots were imaged with an Odyssey Infrared Imaging System (LI-COR Biosciences, Lincoln, NE). The membranes were stripped with Re-Blot Western blot recycling kit (Chemicon International, Temecula, CA) and reprobed.

## Results

### Inhibition of Wnt/β-Catenin Signaling by EA

**Figure 1:**
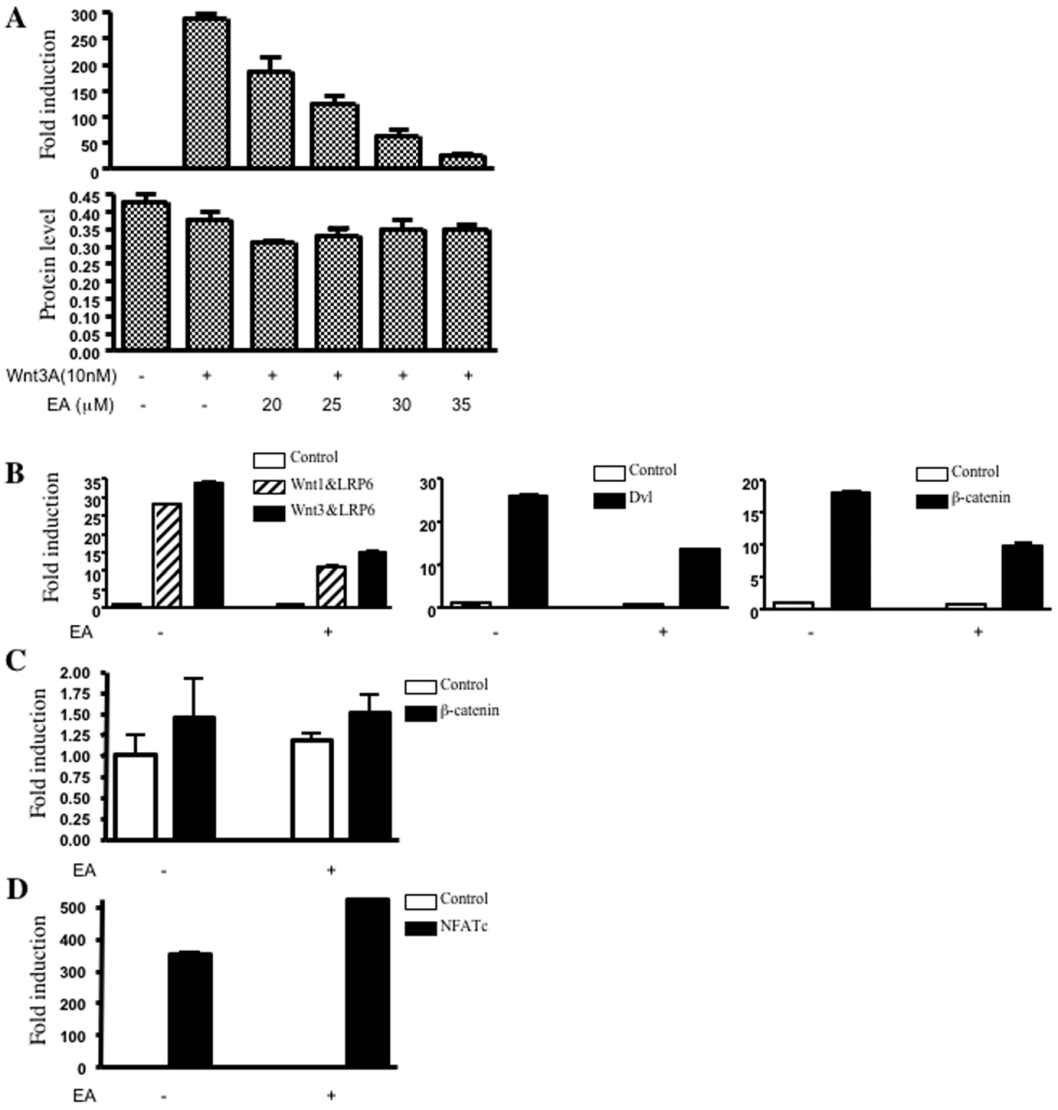
Inhibition of Wnt/β-catenin signaling by EA. (**A**) HEK293 cells carrying a Wnt responsive reporter (Super 8XTOPflash) were treated with Wnt3a (10 nM) and increasing concentrations of EA (20, 25, 30, 35 µM) for 18 hours. Cells were lysed and luciferase activity was quantified. Total protein levels were determined by Bradford Assay and serve as a control for total cell number. (**B**) HEK293 cells were co-transfected with TOPflash reporter construct, along with expression plasmids for Wnt1, Wnt3, LRP6, Dvl and β-catenin as indicated. After transfection for 24 h, the cells were treated with 50 µM EA for another 24 hours, and then luciferase activities were determined. (**C**) HEK293 cells were transfected with FOPflash reporter with or without an expression plasmid for β-catenin. After transfection, the cells were treated with 50 µM EA for another 24 hours. (**D**) HEK293 cells were transfected with NFAT reporter and expression plasmid for NFATc. The cells were treated with 50 µM EA for 24 h, and then harvested, and extracted for determination of luciferase activities. The results are expressed as fold induction of luciferase activity compared to the basal level, and are the means of three experiments ± SEM.

To identify novel antagonists of Wnt/β-catenin signaling, we used a 96-well plate-based TOPflash reporter system to screen the Gen-plus drug library (Microsource) that contains 960 FDA-approved drugs. In this system, transfected Dvl (an upstream activator of the Wnt/β-catenin pathway) stimulated TCF/LEF response elements in the TOPflash reporter gene. In accord with our earlier research, the screen identified several non-steroidal anti-inflammatory drugs (NSAIDs), PPARγ, and RXRα ligands as Wnt antagonists [19]. However, no other compound classes inhibited reporter gene activity, including many known cytotoxic agents. Surprisingly, the screen identified ethacrynic acid (EA), but not other diuretic agents, as a Wnt/β-catenin inhibitor. To further determine the inhibitory effect of EA on Wnt signaling, the stable SuperTOPflash reporter cell line was treated with Wnt3a and increasing concentrations of EA. Wnt3a induced transcriptional activity of the SuperTOPflash reporter 300-fold above the basal levels. EA blocked Wnt3a-induced transcription in a dose-dependent manner (Figure 1A).

To explore possible targets of EA in the Wnt/β-catenin pathway, the TOPflash reporter was activated by Wnt1/LRP6 or Wnt3/LRP6, Dvl and β-catenin, respectively, in transient transfection assays. Treatment with EA reduced Wnt1/LRP6 or Wnt3/LRP6, Dvl, and β-catenin-induced transcription in HEK293 cells (Figure 1B). This action was specific, since the drug had no effect on the FOPflash reporter (Figure 1C). In addition, EA did not block NFATc-mediated transcription from a NFAT reporter (Figure 1D). These results suggest that EA may specifically inhibit Wnt/β-catenin signaling through targeting either β-catenin itself or its downstream factors.

### Selective Cytotoxicity of EA to Chronic Lymphocytic Leukemia (CLL) Cells

**Figure 2:**
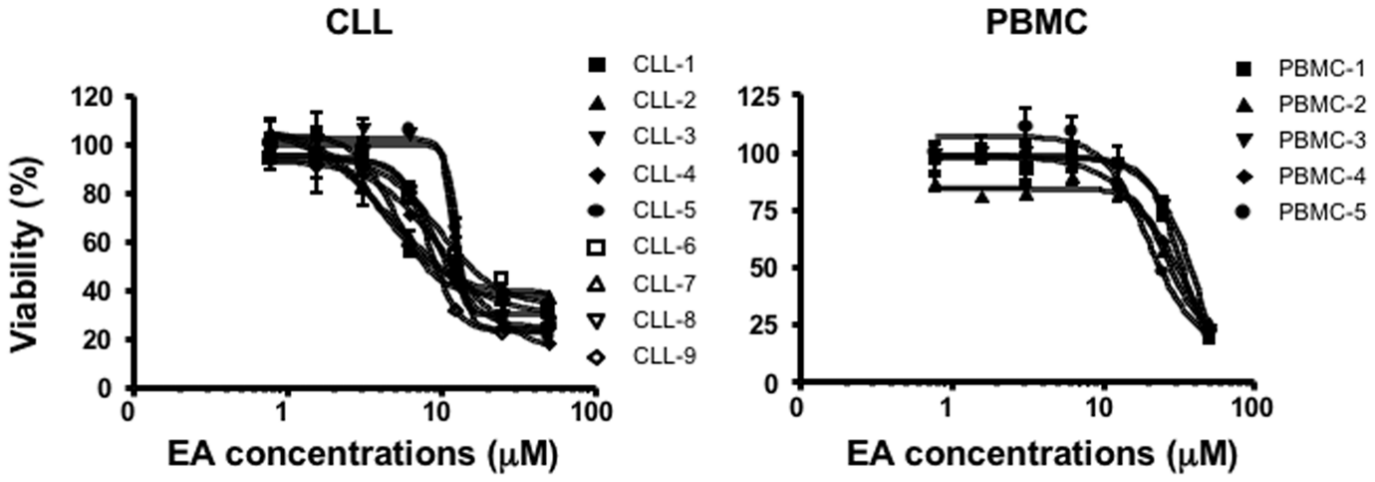
Selective cytotoxicity of EA to CLL cells. Primary CLL cells or normal peripheral blood mononuclear cells (PBMC) were treated with increasing concentrations of EA for 48 h. The cell viability was measured by MTT assay. The control condition was a 2-day incubation of the cells in the medium alone, and the viability expressed as the percentage with respect to this control.

We next tested the cytotoxicity of EA in different tumor cell lines, and in primary CLL cells that are known to have constitutive Wnt activation and very high levels of LEF-1. As shown in Table 1, the mean 50% inhibitory concentration (IC_50_) of EA in these cell lines was in the 40–200 µM range. However, primary CLL cells are highly sensitive to EA. This drug showed selective cytotoxicity with a mean IC_50_ of 8.56+/−3 µM in primary CLL cells, compared to 34.79+/−15.97 µM in normal peripheral blood mononuclear cells (P<0.001) (Table 1 & Figure 2). In addition, we noted that obvious cell death occurred after treatment with EA for 48 hours (data not shown). These findings are in agreement with a previous report by Twentyman et al [14].

**Table 1:** Cytotoxicity of EA in different tumor cell lines and primary CLL cells.

Cell line name	IC_50_-EA (µM)*
Primary CLL cells	8.56±3.0
Normal PBMC	34.79±15.97
LNCap	46
PC3	67
HCT116	58
SW480	68
HT29	56
MCF-7	63
SK-Mel-28	122
HepG2	223
A549	178
U266	90
B16	201
RAMOS	174

* IC_50_ is the mean concentration of drug that reduced cell survival by 50% in at least two experiments. Primary CLL cells were isolated from nine patients. Peripheral blood mononuclear cells (PBMC) were isolated from five normal individuals.

### EA Depresses the Expression of LEF-1, Cyclin D1 and Fibronectin

**Figure 3:**
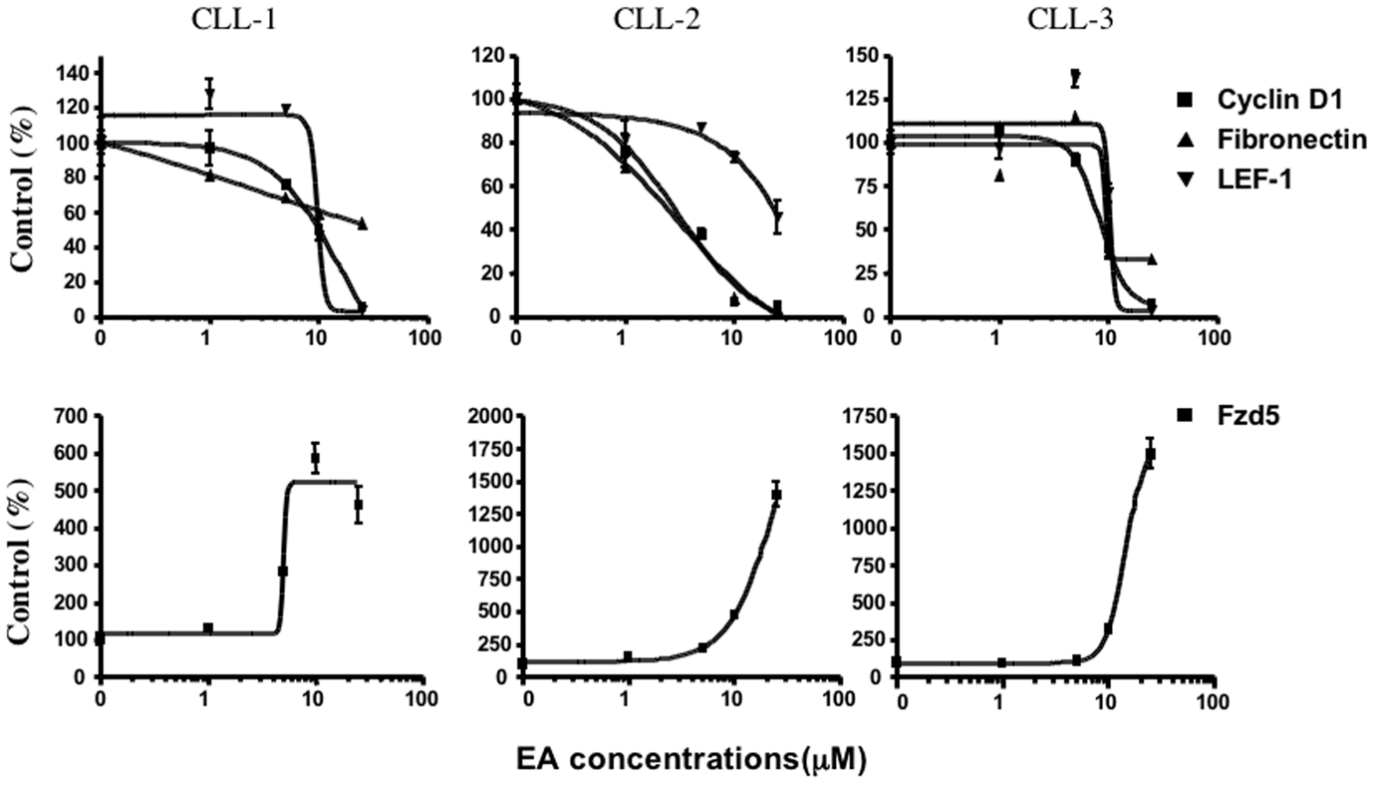
Effect of EA on LEF-1, cyclin D1, fibronectin and Fzd5 expression in CLL cells. CLL cells from three patients were treated with increasing amounts of EA for 16 h. The mRNA levels of LEF-1, cyclin D1, fibronectin and Fzd5 were compared by real-time PCR. Total RNA input was normalized based on the concentration of 18S RNA.

To assess the inhibitory effects of EA on Wnt/β-catenin signaling in CLL cells, real-time PCR was employed to detect the expression of some Wnt target genes. LEF-1, cyclin D1 and fibronectin are established target genes of the Wnt/β-catenin pathway [23], [24], [25], [26], [27]. The expression of Fzd5 was also detected in our experiments. Fzd5 is not a target gene of Wnt/β-catenin signaling. To determine the ability of EA to alter LEF-1, cyclin D1, fibronectin and Fzd5 transcript expression, CLL cells from three patients were treated with the drug for 16 h, and then analyzed by real-time PCR. Total RNA input was normalized to the concentration of 18S RNA. As shown in Figure 3, EA decreased LEF-1, cyclin D1 and fibronectin mRNA expression in a concentration-dependent fashion in CLL cells. Interestingly, EA showed dose-dependent enhancement of Fzd5 expression (Figure 3). It is unclear how EA enhances the expression of Fzd5.

### EA Directly Interacts with LEF-1 and Destabilizes the LEF-1/β-Catenin Complex

**Figure 4:**
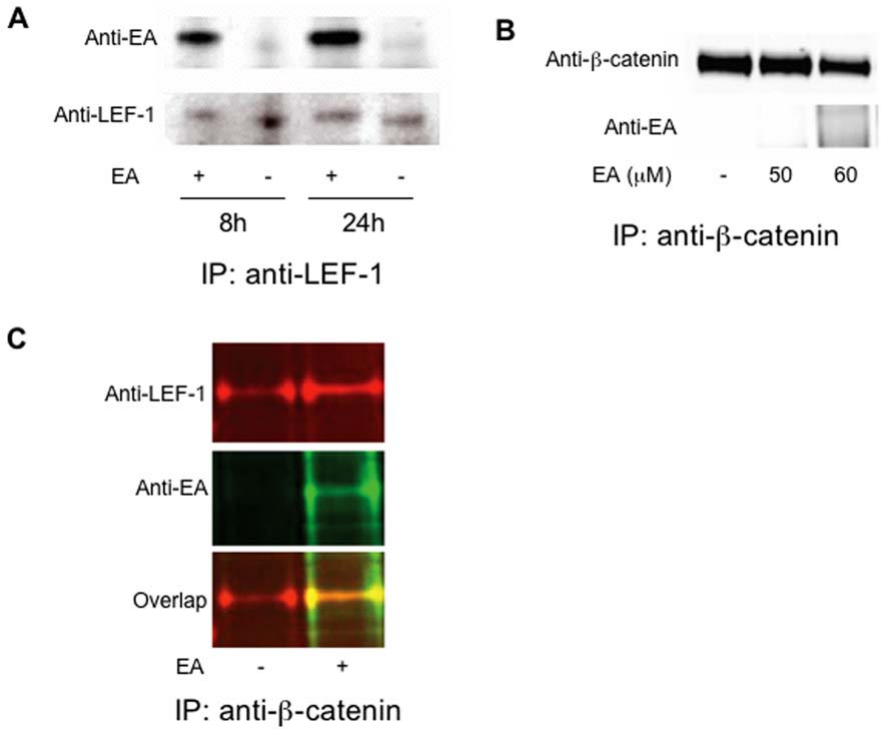
EA binds to LEF-1 in primary CLL and SW480 cells. (**A**) lysates from CLL cells exposed to 10 µM EA for 8 h or 24 h were immunoprecipitated with anti-LEF-1 antibody. The immune complexes were analyzed by immunoblotting with anti-LEF-1 and anti-EA antibodies. (**B**) SW480 cells were treated with indicated amounts of EA for 16 h. Cell lysates were immunoprecipitated with anti-β-catenin antibody (Santa Cruz Biotechnology, Santa Cruz, CA). The immune complexes were analyzed by immunoblotting with anti-EA and anti-β-catenin antibodies. (**C**) SW480 cells were exposed to 50 µM of EA for 16 h and cell lysates were immunoprecipitated with anti-β-catenin antibody. The proteins in the immunoprecipitates were resolved by SDS-PAGE, transferred, and probed with indicated antibodies. The LEF-1 protein band, as confirmed by reactivity with LEF-1 specific antibody, stained positive with the anti-EA antibody only in the drug treated samples.

Since the initial results indicated that EA may target either β-catenin itself or its downstream factors, we next explored whether the drug could directly interact with any component of the β-catenin complex. The unsaturated ketone in EA can undergo Michael addition with free thiols. To detect such covalent modifications, we prepared antibody to EA conjugated to an irrelevant protein carrier, and used the antibody to probe CLL cells exposed to the drug by immunoblotting. As expected, EA could interact with multiple proteins in CLL extracts, but did not bind detectably to β-catenin itself (data not shown). However, the antibody to EA consistently recognized a 47 kD protein consistent with the approximate size of LEF-1 (data not shown). To determine whether EA could indeed directly interact with LEF-1, CLL cells that had been treated with the drug were lysed, immunoprecipitated with anti-LEF-1 antibody, and probed in immunoblots using the anti-EA antibody (Figure 4A). The immunoprecipitated LEF-1 protein from CLL lysates treated with EA at 10 µM for 8 h and 24 h reacted strongly with the anti-EA antibody.

To test whether EA could similarly interact with LEF-1 in other tumor cells, a human colon cancer cell line SW480 was tested. It has been demonstrated that LEF-1 is constitutively associated with β-catenin in SW480 cells [28]. If EA inhibits Wnt/β-catenin signaling through directly interacting with LEF-1, one would expect that the drug should bind to LEF-1 in the LEF-1/β-catenin complex. Hence, an anti-β-catenin antibody was used to pull down the β-catenin complex in a co-immunoprecipitation experiment. SW480 cells were treated with the indicated amounts of EA in Figure 4 for 16 hours. Cell lysates were immunoprecipitated with anti-β-catenin antibody. The precipitated β–catenin could not be detected by anti-EA antibody (Figure 4B). However, the anti-β-catenin antibody pulled down LEF-1 protein and EA (Figure 4C), indicating that the drug may directly bind to LEF-1 in the LEF-1/β-catenin complex in SW480 cells.

**Figure 5:**
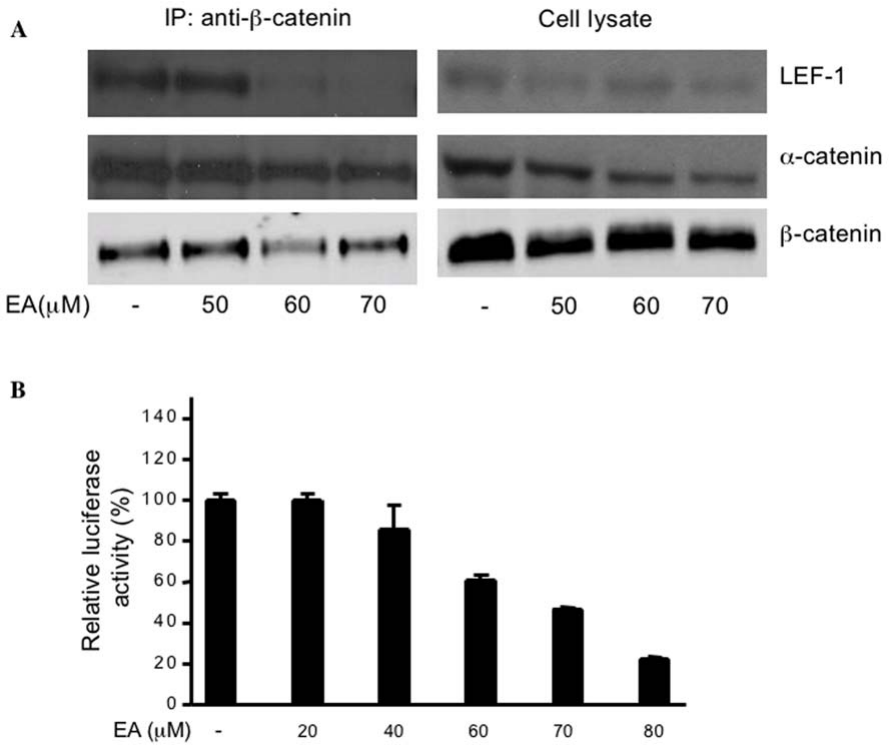
EA destabilizes the LEF-1/β-catenin complex. (**A**) SW480 cells were treated with increasing amounts of EA for 16 h. Cells were lysed, and IP was completed with anti-β-catenin monoclonal antibody. The immune complexes were analyzed by immunoblotting with anti-LEF-1, anti-α-catenin and anti-β-catenin antibodies. (**B**) EA inhibits Wnt/β-catenin signaling in SW480 cells. SW480 cells were transfected with TOPflash reporter and control plasmid pCMXβgal. After transfection for 24 h, cells were treated with increasing concentrations of EA for another 24 h as indicated. Cells were then harvested and luciferase values were determined. The results are expressed as relative luciferase activity (%) normalized to a β–galactosidase control.

To determine the consequences of EA modification of the LEF-1/β-catenin complex, SW480 cells were treated with increasing concentrations of the drug, and after 16 hours, the β-catenin complex was pulled down using antibody against β-catenin. Immunoblot analysis demonstrated that EA decreased LEF-1 levels in the β-catenin complex in a concentration-dependent manner (Figure 5A). However, EA had little effect on α-catenin levels. This result suggests that EA binding to LEF-1 may lead to destabilization of the LEF-1/β-catenin complex. In whole cell lysate, we observed that EA treatment decreased the level of α-catenin. It is possible that EA may downregulate the expression of α-catenin via some as yet unknown mechanism.

We next checked EA effect on TOPflash activity in SW480 cells. EA exhibited dose-dependent inhibition at concentrations equal to and above 60 µM, the dose required to destabilize the LEF-1/β-catenin complex (Figure 5B). This result suggests that EA may inhibit LEF-1-mediated transcription through destabilization of the LEF-1/β-catenin complex in colorectal cancer cells.

### N-Acetyl-L-Cysteine (NAC) Prevents EA-Mediated Effects on the Wnt/β-Catenin Pathway and on CLL Survival

**Figure 6:**
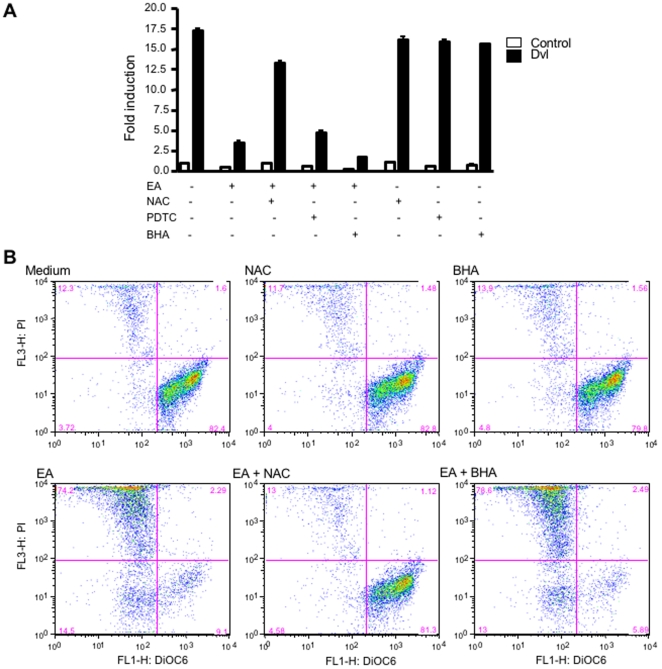
N-acetyl-L-cysteine (NAC) prevents EA-mediated effects on the Wnt/β-catenin pathway and on CLL survival. (**A**) prevention of EA-mediated inhibition of Wnt/β-catenin signaling by free thiols. HEK293 cells were co-transfected with TOPflash reporter vector, and with a Dvl vector to activate signaling. The transfected cells were treated with 70 µM EA, 1 mM NAC, 100 µM PDTC, or 100 µM BHA, as indicated in the figure. After 24 h incubation, cell extracts were assayed for luciferase activities. (**B**) rescue of CLL cells from EA-induced apoptosis by NAC. Primary CLL cells were treated with 3 µM EA, 1 mM NAC, 100 µM BHA or combined treatment as indicated. After treatment for 48 h, the cells were stained with DiOC_6_ and PI and analyzed by flow cytometry. Note that NAC, but not other anti-oxidants, protected the CLL cells from EA-induced apoptosis.

Although previous reports showed cytotoxicity of EA in different tumor cell lines, the mechanism of cell killing was unknown [14], [17]. To examine if the inhibition of Wnt/β-catenin signaling by EA is mediated by modulation of thiols, or by increased oxidative stress as a result of GST inhibition, cells that had been co-transfected with the TOPflash reporter and the Dvl expression plasmids were treated with EA and various antioxidants (NAC, BHA and PDTC). NAC, an antioxidant containing a reactive free thiol group, significantly prevented EA-induced inhibition of Wnt/β-catenin signaling. In contrast, neither BHA, which scavenges reactive oxygen species (ROS), but does not have a free thiol, nor PDTC, which does react efficiently with EA, did not reverse the Wnt inhibition (Figure 6A). Consistent with this result, neither a glutathione synthesis inhibitor (buthionine sulfoximine, BSO) nor a superoxide dismutase inhibitor (2-methoxyestradiol), blocked Wnt/β-catenin signaling in the cell-based system (data not shown).

To test whether NAC has the ability to protect CLL cells from EA-induced apoptosis, a highly EA-sensitive CLL sample was treated with EA alone or combined with NAC for 48 hours. The result showed that NAC (1 mM) significantly protected CLL cells from EA-induced apoptosis, but the free radical scavenger BHA (100 µM) had no effect (Figure 6B), suggesting that the inhibition of Wnt/β-catenin signaling and apoptosis in CLL by EA is not mediated by the generation of oxygen radicals.

## Discussion

The Wnt signaling pathway has been shown to play a critical role in the early phases of B lymphocyte development, but is thought to be less important for the survival of normal mature B cells [29]. Although CLL cells have the morphological characteristics of mature B lymphocytes, they frequently over-express Wnt pathway genes associated with pro-B or pre-B cells, including Wnt3, Wnt16, the orphan Wnt receptor ROR1, and the LEF-1 transcription factor [10], [11], [12], [30], [31]. The immature pro-B cells from LEF-1 deficient mice display increased sensitivity to apoptosis [29], although the exact mechanism is unclear. We hypothesized that interference with the Wnt/β-catenin/LEF-1 pathway might sensitize CLL cells to apoptosis. The results of the present experiments support this supposition.

To identify potential pharmacologic antagonists of Wnt signaling, a 960-member library of known drugs was screened using a cell-based TOPflash reporter gene assay. Among the few drugs that blocked the Wnt reporter gene activity, at concentrations that did not affect a control reporter gene, was the loop diuretic ethacrynic acid (EA). The antagonism was not attributable to non-specific toxicity of EA. Moreover, neither DNA damaging agents nor anti-metabolites used in cancer therapy displayed inhibitory effects in this Wnt dependent system. Experiments with the cell-based reporter system demonstrated that EA inhibited Wnt/β-catenin signaling mediated not only by Wnt3a, but also by Wnt/LRP6, Dvl and β-catenin, respectively, suggesting that the drug may target either β-catenin itself or its downstream factors. Subsequent studies showed that EA could not bind to β-catenin. Instead, EA directly interacted with LEF-1, and induced the destabilization of the LEF-1/β-catenin complex. LEF-1 has been shown to have free thiol groups that are required for maintenance of its structure [32]. In cells whose survival depends upon LEF-1 activity, modification of these thiols by EA may be lethal.

Experiments with real-time PCR demonstrated that treatment with EA caused a dose-dependent decline in the expression of three Wnt target genes, LEF-1, cyclin D1 and fibronectin, which reflects EA inhibition of Wnt/β-catenin signaling in CLL cells. However, EA enhanced the expression of Fzd5 in a concentration-dependent manner. Fzd5 is a member of Frizzled receptor family. It has been shown to activate both canonical and noncanonical Wnt pathways through binding Wnt proteins such as Wnt5a, Wnt7a and Wnt11 [33], [34], [35]. Interestingly, a recent study demonstrated that apoptotic agents imatinib and etoposide could also up-regulate Fzd5 expression in the myeloid cell lines K562 and HL60 [36]. We speculate that the increased expression of Fzd5 might correlate with an apoptotic process. Further studies are needed to confirm our speculation.

EA is a loop diuretic drug that was formerly widely used, and demonstrated an excellent safety profile, despite its α, β-unsaturated ketone, that can modify free thiol residues of proteins. Our previous studies demonstrated that reduction of the C-C double bond in EA abrogated its ability both to block Wnt signaling and to impair CLL survival [18]. Here we showed that N-acetyl-L-cysteine (NAC) significantly prevented the EA-mediated inhibition of the Wnt/β-catenin pathway and EA-induced apoptosis in CLL cells. NAC is a known precursor and upregulator of GSH. It may mediate its functions by formation of GSH-conjugates that can be removed by the multidrug resistance pump or by directly reversing EA-alkylated cysteine residues. To determine whether depletion of GSH is associated with the inhibition of Wnt/β-catenin signaling, buthionine sulfoximine (BSO) was used to deplete GSH in cells. Treatment with BSO did not inhibit Wnt/β-catenin signaling (data not shown), suggesting that the depletion of GSH is probably not responsible for EA effect on Wnt/β-catenin signaling. Since EA is able to increase cellular oxidative stress through inhibiting GST, we also tested the effect of two free radical scavengers, BHA and PDTC, on EA-mediated inhibition of Wnt/β-catenin signaling. Both scavengers could not prevent EA-induced inhibition (Figure 6A). Moreover, we did not observe any significant increase in the levels of O_2_ **^−^** or H_2_O_2_ in CLL cells treated with EA (results not shown). These results indicate that increased oxidative stress is not responsible for the selective killing of CLL cells by EA. Similarly, Aizawa et al. reported that EA could induce cell death in a human colon cancer cell line (DLD-1) via an oxidative stress independent mechanism [17].

Our experiments demonstrated potent cytoxicity of EA in primary CLL cells with IC_50_ of 8.56+/−3 µM, while the IC_50_ for EA inhibition of Wnt3A-induced transcription in HEK293 is about 25 µM, and the EA concentration required to destabilize the LEF-1/β-catenin complex is at least 60 µM. This difference reflects the cell type specific effect on EA sensitivity. CLL cells are known to have low levels of GSH that can react with EA [37]. There is also differential dependence of cell survival on LEF-1/β-catenin signaling, which is probably critical for CLL, but not many other cell types.

Compared to cultured tumor cell lines and to normal peripheral blood mononuclear cells, primary CLL cells were 5–50 fold more sensitive to the cytotoxic effects of EA. It is tempting to speculate that the sensitivity may be related to the high levels in CLL of LEF-1 and its downstream effectors such as ROR1, compared to most other cell types [12], [31]. Accordingly, LEF-1 could be a critical target for chemotherapy in CLL cells.

NF*-*κB signaling is another anti-apoptotic pathway which is constitutively activated in CLL cells, and may render them resistant to normal mechanisms of apoptosis [38], [39]. Previous studies have revealed that inhibition of NF-κB by drugs induces apoptosis of CLL cells [39], [40]. Han et al. reported that EA could inhibit activation of the NF-κB pathway at multiple steps [41]. Thus, inhibition of NF-κB may synergize with Wnt antagonism to impair CLL survival.

In addition, other signaling pathways may also contribute to the cytotoxic effects of EA on various cell types. It has been reported that the mitogen activated protein kinase (MAPK) pathway may be involved in EA-induced cell death [17], but MAPK inhibitors are not generally cytotoxic to CLL cells. A recent study showed that EA and its butyl ester prodrug induced apoptosis in leukemia cells through a hydrogen peroxide–mediated pathway, although in CLL cells ant-oxidants other than N-acetyl-L-cysteine did not abrogate either the drug's toxicity or its ability to block Wnt signaling [42]. Because EA can bind to any free thiol, it is likely that the mechanism of EA-induced cell death is variable and will depend upon cell type and context. The high sensitivity of CLL cells to EA may be attributed to its multiple effects on both Wnt and NF-κB signaling.

Recently, we could show a significant induction of apoptosis by EA and ciclopiroxolamine (cic) in lymphoma and myeloma cells [43]. Our data suggest that EA and cic can inhibit Wnt/β-catenin signalling in lymphoma and myeloma cell lines. Our results are in accordance with a recent report [44] that the canonical Wnt signalling pathway is activated in multiple myeloma through constitutively active β-catenin.

In summary, these experiments suggest that EA selectively suppresses CLL survival in part due to inhibition of Wnt/β-catenin signaling. Antagonizing Wnt signaling in CLL with EA or related drugs may represent an effective treatment of this disease. O'Dwyer and colleagues reported a phase I trial of EA in patients with advanced solid tumors [45], [46]. The toxicities associated with the diuretic effect were easily managed with proper monitoring. The maximum plasma concentrations of EA ranged from 2.66 to 9.38 µg/ml (8.8–30.9 µM) after i.v. administration. Moreover, their results suggested that continuous i.v. infusion can be used to achieve and sustain plasma concentrations greater than 1 µg/ml (3.3 µM) for up to 3 hr [45]. In this study, we demonstrated the selective cytotoxicity of EA in primary CLL cells with a mean IC_50_ of 8.56+/−3 µM, which can be achieved in patients. In addition, our preliminary results have shown that treatment with 3 µM EA enhanced fludarabine-mediated apoptosis of CLL cells (data not shown). These results suggest that EA, by inhibition of the Wnt/β-catenin pathway, compromised an important survival signal in CLL cells and increased their vulnerability to cell killing induced by chemotherapeutic agents. Therefore, there is a need to evaluate the therapeutic potential of EA alone or combined with other cytotoxic agents, such as fludarabine, in CLL patients.
